# A systems biology approach to pathogenesis of gastric cancer: gene network modeling and pathway analysis

**DOI:** 10.1186/s12876-023-02891-4

**Published:** 2023-07-24

**Authors:** Negar Mottaghi-Dastjerdi, Abozar Ghorbani, Hamed Montazeri, Pietro Hiram Guzzi

**Affiliations:** 1 Department of Pharmacognosy and Pharmaceutical Biotechnology, School of Pharmacy, Iran University of Medical Sciences, Tehran, Iran; 2 Nuclear Agriculture Research School, Nuclear Science and Technology Research Institute (NSTRI), Karaj, Iran; 3 Department of Surgical and Medical Sciences, University “Magna Græcia” of Catanzaro, Catanzaro, Italy

**Keywords:** Biological networks, Differentially expressed genes, Gastric cancer, Gene ontology, Kyoto Encyclopedia of Genes and Genomes, Promoter analysis, Protein–protein interactions

## Abstract

**Background:**

Gastric cancer (GC) ranks among the most common malignancies worldwide. This study aimed to find critical genes/pathways in GC pathogenesis.

**Methods:**

Gene interactions were analyzed, and the protein–protein interaction network was drawn. Then enrichment analysis of the hub genes was performed and network cluster analysis and promoter analysis of the hub genes were done. Age/sex analysis was done on the identified genes.

**Results:**

Eleven hub genes in GC were identified in the current study (ATP5A1, ATP5B, ATP5D, MT-ATP8, COX7A2, COX6C, ND4, ND6, NDUFS3, RPL8, and RPS16), mostly involved in mitochondrial functions. There was no report on the ATP5D, ND6, NDUFS3, RPL8, and RPS16 in GC. Our results showed that the most affected processes in GC are the metabolic processes, and the oxidative phosphorylation pathway was considerably enriched which showed the significance of mitochondria in GC pathogenesis. Most of the affected pathways in GC were also involved in neurodegenerative diseases. Promoter analysis showed that negative regulation of signal transduction might play an important role in GC pathogenesis. In the analysis of the basal expression pattern of the selected genes whose basal expression presented a change during the age, we found that a change in age may be an indicator of changes in disease insurgence and/or progression at different ages.

**Conclusions:**

These results might open up new insights into GC pathogenesis. The identified genes might be novel diagnostic/prognostic biomarkers or potential therapeutic targets for GC. This work, being based on bioinformatics analysis act as a hypothesis generator that requires further clinical validation.

## Introduction

Gastric cancer (GC) is the fifth most common cancer and the fourth cause of cancer-related death worldwide, with more than 1 million new cases in 2020 and an estimated 769,000 deaths, equal to one out of each 13 deaths worldwide [1]. Although the association between different risk factors and the development of GC has been investigated in different studies [2], the specific molecular network mechanisms have not been fully introduced. Therefore, elucidating the molecular mechanisms involved in GC pathogenesis may help to find targets for early detection and classification and prolong patient survival.

Carcinogenesis is a complicated process involving several genetic and epigenetic alterations [3]. Adenocarcinoma is the most common type of GC, which has two subtypes, the diffuse type, and the intestinal type, with different molecular characteristics [4]. There are many investigations on the epigenetic and genetic alterations in different types of GC, involving alterations in cell cycle regulators, tumor suppressor genes, DNA repair genes, and oncogenes [5]. The exploration of the genes which are dysregulated in different pathways may be beneficial in elucidating the molecular pathophysiological mechanisms underlying carcinogenesis and, therefore, may help to develop new treatment strategies. Recently, gene and network analysis using high-throughput methods have been used as hopeful tools with several clinical applications, including cancer detection and classification, as well as patient response to the treatment and the prognosis of the disease [6, 7]. Using systems biology and systems pharmacology approaches can provide a better understanding of mechanisms and detect gene signatures for precision and personalized medicine [8]. These types of systems-level insights can be used to design more accurate in silico models of biological circuits leading to cells and tumor responses [9, 10].

There are many investigations on alterations of the genes in GC. However, they are not still enough to provide an elucidated picture of the molecular pathogenesis of GC [11]. Therefore, along with these studies, and to provide more clues into the mysterious molecular pathogenesis of GC, we previously studied the gene expression profile of GC using the suppression subtractive hybridization (SSH) method. We introduced the overexpressed genes in GC [12–14]. The present study uses bioinformatics analysis to investigate the genes involved in the pathogenesis of GC introduced in our previous studies and investigates the important Gene Ontology (GO) terms, Kyoto Encyclopedia of Genes and Genomes (KEGG) pathways, and protein–protein interaction (PPI) networks, with a specific focus on potential gene hubs which have possible roles in the gastric carcinogenesis. In particular, the current study tries to shed light on changes in disease progression and comorbidities related to age.

## Methods

### Selection of the genes involved in gastric carcinogenesis

In our previous studies [12–14], we used the SSH method to identify the overexpressed genes in gastric tumor tissue compared to normal tissue, and finally, we identified thirteen genes as the overexpressed genes in GC based on the SSH method. A list of these genes is provided in Table 1 and these are the genes that are used in our current study for network analysis.

**Table 1:** List of the genes used in this study as the overexpressed genes identified in GC by the SSH method [12–14]

**#**	**Gene name**	**Gene ID**	**Cytogenetic location**	**Description**
1	RPL18A	6142	19p13	60S ribosomal protein L18a
2	TSPAN8	7103	12q14.1–q21.1	Tetraspanin 8
3	COII	4513	MT	Cytochrome c oxidase subunit 2 (COII)
4	ND4	4538	MT	NADH dehydrogenase, subunit 4
5	ATP6	4508	MT	Mitochondrially encoded ATP synthase 6
6	EIF4A1	1973	17p13	Eukaryotic translation initiation factor 4A1
7	RNASEH2B	79621	13q14.3	Ribonuclease H2, subunit B; AicardiGoutieres syndrome 2 protein; RNase H2 subunit B
8	SEC13	6396	3p25-p24	Protein SEC13 homologue
9	VAT-1	10493	17q21.31	Vesicle amine transport protein-1
10	*MT-RNR2L1 (HN1)*	100462977	17p11.2	MT-RNR2 like 1 (pseudogene)
11	*MT-RNR2L3 (HN3)*	100462983	20q13.31	MT-RNR2 like 3 (pseudogene)
12	*MT-RNR2L6 (HN6)*	100463482	7q34	MT-RNR2 like 6 (pseudogene)
13	*MT-RNR2L10 (HN10)*	100463488	Xp11.21	MT-RNR2 like 10 (pseudogene)

### Reconstruction of genes and PPI networks and the hub analysis

STRING is a database of predicted and known interactions of proteins. To evaluate the interactions among the genes listed in Table 1, these genes were analyzed in the web-based application of STRING version 10 (http://string-db.org) [15] with a minimum required interaction score of 0.15 (low confidence), and the PPI list was prepared. Subsequently, the PPI list was imported into Cytoscape (version 3.9.1), an open-source platform for the visualization of molecular interaction networks and data integration. *CytoHubba* software (version 0.1) was used to identify hub proteins between all nodes [16]. Four topological analysis methods, including MCC, Degree, DMNC, and MNC, were used to evaluate the significance of the nodes in the biological network. Four high-ranked proteins were introduced as the hub nodes from the analysis by each method. Subsequently, the subnetwork showing the interactions among the hub nodes was drawn using the *CytoHubba* plugin.

### Gene Ontology and pathway enrichment analysis of the hub genes

The enrichment analysis of subnetwork genes of the hub genes was done based on the KEGG and GO, including molecular function (MF), cellular components (CC), and biological process (BP) at the web-based application of STRING [15].

### Cluster analysis of the network

CytoCluster (version 2.1.0) was used for the clustering of network nodes. The IPCA (Identifying Protein Complex Algorithm) algorithm was used for cluster analysis of the subnetwork. This algorithm is density-based, which identifies dense subgraphs in protein interaction networks. IPCA determines the weight of an edge by calculating the common neighbors of its connected nodes and calculates each node’s weight through a sum up of the weights of its incident edges. [17]. The threshold was set to 10, and the genes of each cluster were further analyzed in STRING version10 [15] to find the KEGG pathways in which these genes are involved.

### Promoter motif analysis of hub genes

The 1 kbp upstream flanking regions (UFRs) of hub genes were extracted from Ensembl BioMart web services (https://asia.ensembl.org/info/data/biomart/index.html). Conserved motifs on the sequences were identified using MEME Suite (version 5.4.1) (meme.nbcr.net/meme/intro.html) [18] with its default parameters except for the threshold P- and E-values of < 0.01 and < 0.0001, respectively. Tomtom (version 5.4.1) tool (http://meme-suite.org/tools/tomtom) [19] was used to remove redundant motifs and identify known CRE based on the motif database of Human (homo sapiens) DNA (HOCOMOCO Human (v11 Full) with the threshold P- and E-values of < 0.01 and < 0.0001, respectively. GoMo tool (http://meme-suite.org/tools/gomo) was also used to find potential roles for motifs [20].

### Age–tissue analysis

For each gene selected in the previous steps, we downloaded expression data from the GTEx portal [21] accessed on September 25th 2022. For each sample, we also downloaded age information. Age information is grouped into six intervals: 20–29, 30–39, 40–49, 50–59, 60–69 ad 70–79. We grouped genes by using these labels and for each group, we considered the median value. First, filtered those genes whose average values are increasing or decreasing considering such intervals. Moreover, since the prevalence of gastric cancer significantly increases around 60 years we grouped genes into two classes, 19–59 and 60–79 and we compared the average values between these classes.

## Results

### Reconstruction of genes and PPI networks and the hub analysis

**Fig. 1:**
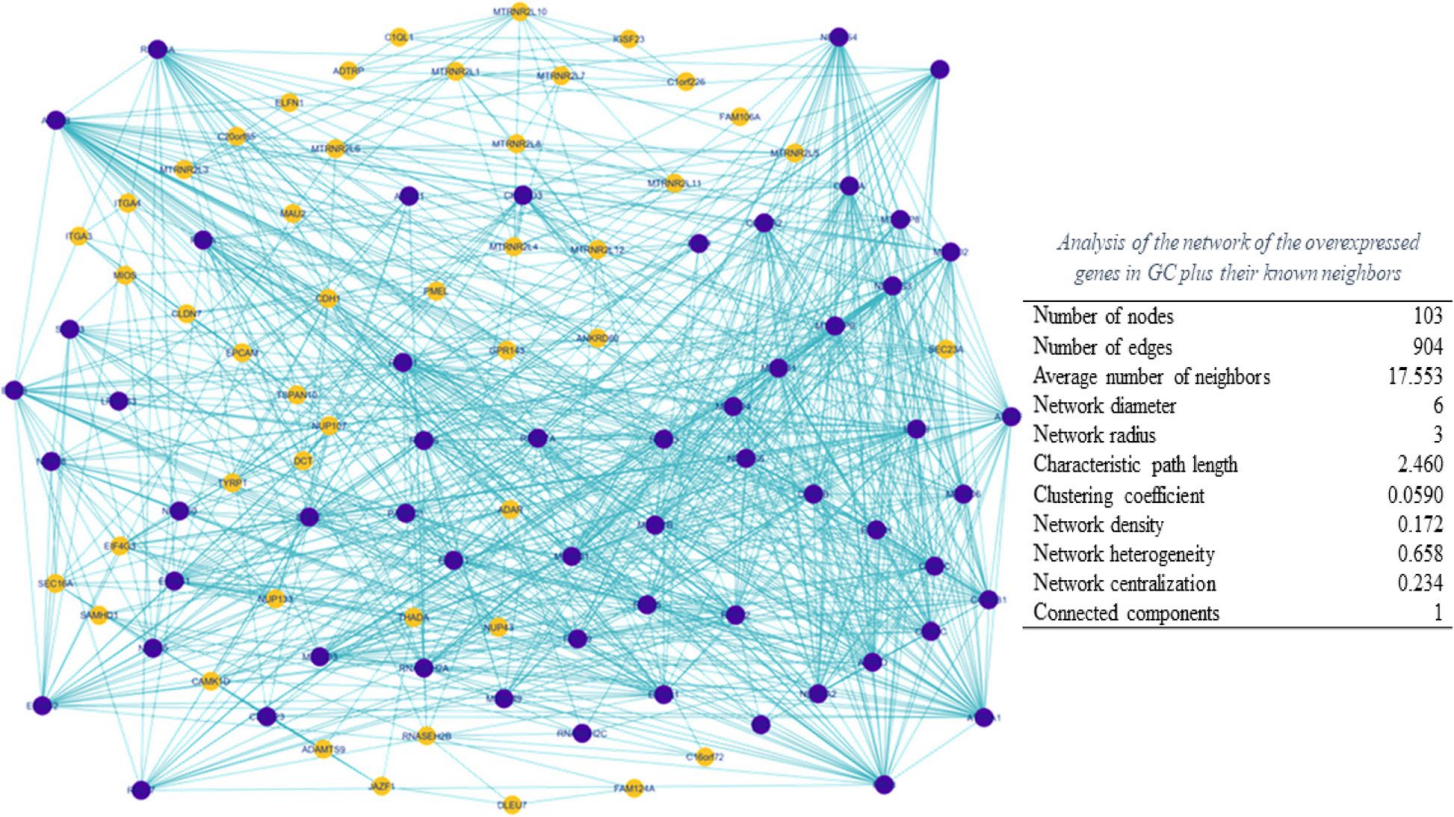
Network of the overexpressed genes in Gastric Cancer plus their known neighbors based on data provided in our previous studies [12–14] using Cytoscape software. The subnetwork genes are colored in blue

**Fig. 2:**
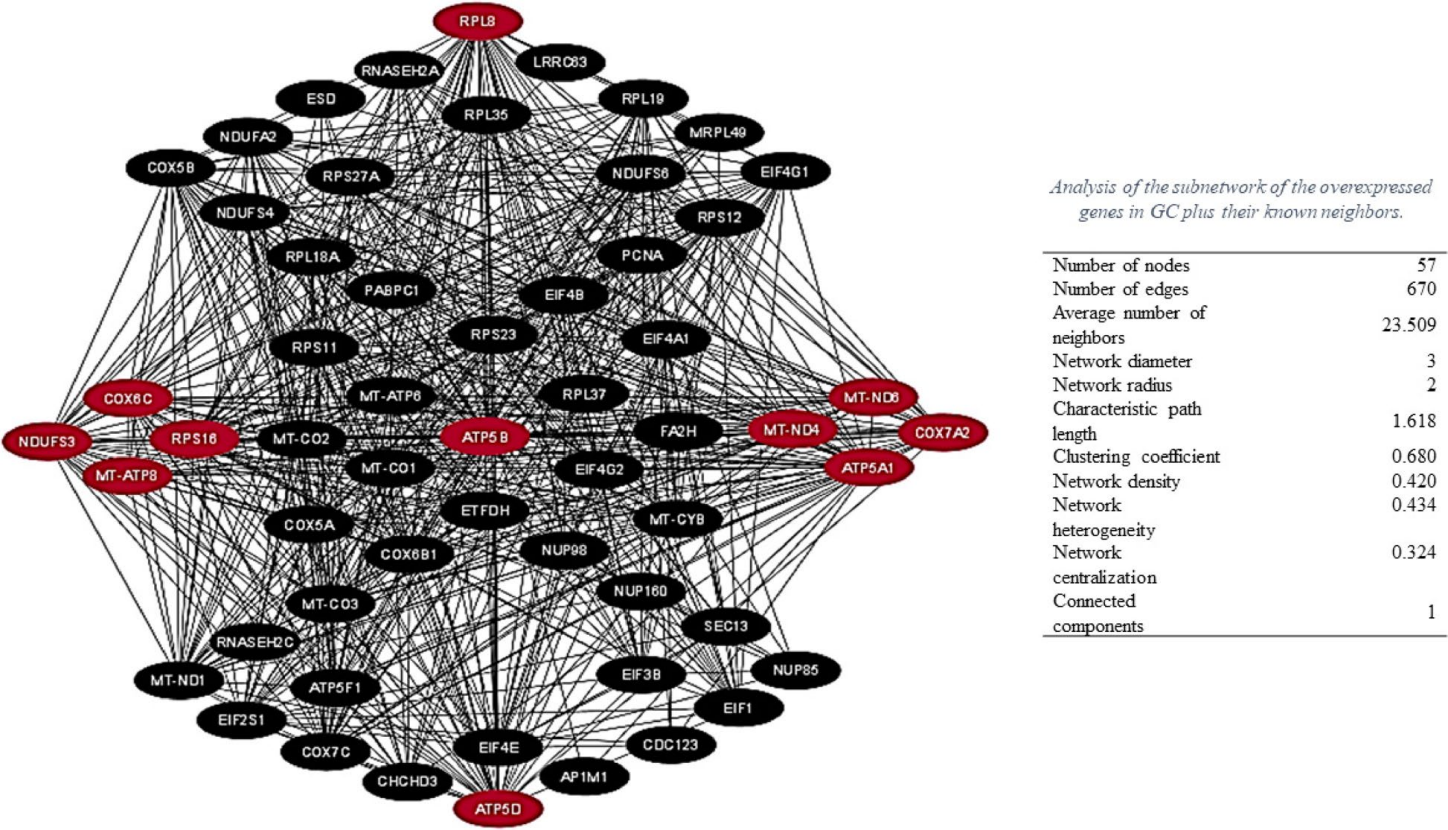
Subnetwork of hub genes the overexpressed genes in Gastric Cancer plus their known neighbors based on data provided in our previous studies [12–14] using the *CytoHubba* App

In the present study, bioinformatics analysis of differentially expressed genes (DEGs) was performed in GC based on the identified genes from our previous studies [12–14]. The reconstructed gene and PPI networks are shown in Fig. 1. The hub analysis resulted in the identification of 11 genes with the most interactions (Table 2). The subnetwork prepared by these identified hub genes is illustrated in Fig. 2**.**

**Table 2:** List of hub genes identified by using *CytoHubba*. *CytoHubba* software (version 0.1) was used to draw the PPI network and identify hub proteins between all nodes. The hub analysis resulted in the identification of 11 genes with the most interactions

**#**	**Node**	**Gene ID**	**Ensembl**	**Gene Description**	**Rank**	**Ranking Method**	**Other names**	**Location**
**1**	ATP5A1	498	ENSG00000152234	ATP synthase F1 subunit alpha	1	MCC, MNC, Degree	OMR; ORM; ATPM; MOM2; ATP5A; hATP1; ATP5F1A; MC5DN4; ATP5AL2; COXPD22; HEL-S-123 m	18q21.1
**2**	ATP5B	506	ENSG00000110955	ATP synthase F1 subunit beta	2	MCC, MNC, Degree	ATP5F1B; ATPMB; ATPSB; HEL-S-271	12q13.3
**3**	NDUFS3	4722	ENSG00000213619	NADH: ubiquinone oxidoreductase core subunit S3	3	MCC	CI-30; MC1DN8	11p11.2
**4**	COX6C	1345	ENSG00000164919	Cytochrome c oxidase subunit 6C	4	MCC	-	8q22.2
**5**	MT-ND6	4541	ENSG00000198695	mitochondrially encoded NADH dehydrogenase 6	1	DMNC	MTND6; ND6	MT (non-nuclear)
**6**	MT-ATP8	4509	ENSG00000228253	mitochondrially encoded ATP synthase 8	2	DMNC	ATPase8; MTATP8; ATP8	MT (non-nuclear)
**7**	MT-ND4	4538	ENSG00000198886	mitochondrially encoded NADH dehydrogenase 4	3	DMNC	MTND4; ND4	MT (non-nuclear)
**8**	COX7A2	1347	ENSG00000112695	cytochrome c oxidase subunit 7A2	4	DMNC	VIIAL; COX7AL; COX7AL1; COXVIIAL; COXVIIa-L	6q14.1
**9**	RPL8	6132	ENSG00000161016	ribosomal protein L8	2	MNC, Degree	L8	8q24.3
**10**	ATP5D	513	ENSG00000099624	ATP synthase F1 subunit delta	2	MNC	ATP5F1D; MC5DN5	19p13.3
**11**	RPS16	6217	ENSG00000105193	ribosomal protein S16	2	Degree	S16	19q13.2

As shown in Table 2, four genes encode different subunits of the ATP synthase, including ATP5A1, ATP5B, ATP5D, and MT-ATP8. Three identified genes in this study (Table 2), including NDUFS3, MT-ND4, and MT-ND6, encode subunits of complex I (CxI). Among the identified genes in this study (Table 2), two genes, including COX7A2 and COX6C, encode cytochrome c oxidase (Cytc) subunits. Finally, two ribosomal proteins, RPL8 and RPS16 (Table 2), were also identified in the present study. RPL8 encodes ribosomal L8 protein, which is one of the components of the 60S subunit [22]. RPS16 encodes ribosomal protein S16 [23].

### Gene Ontology and pathway enrichment analysis of genes

**Fig. 3:**
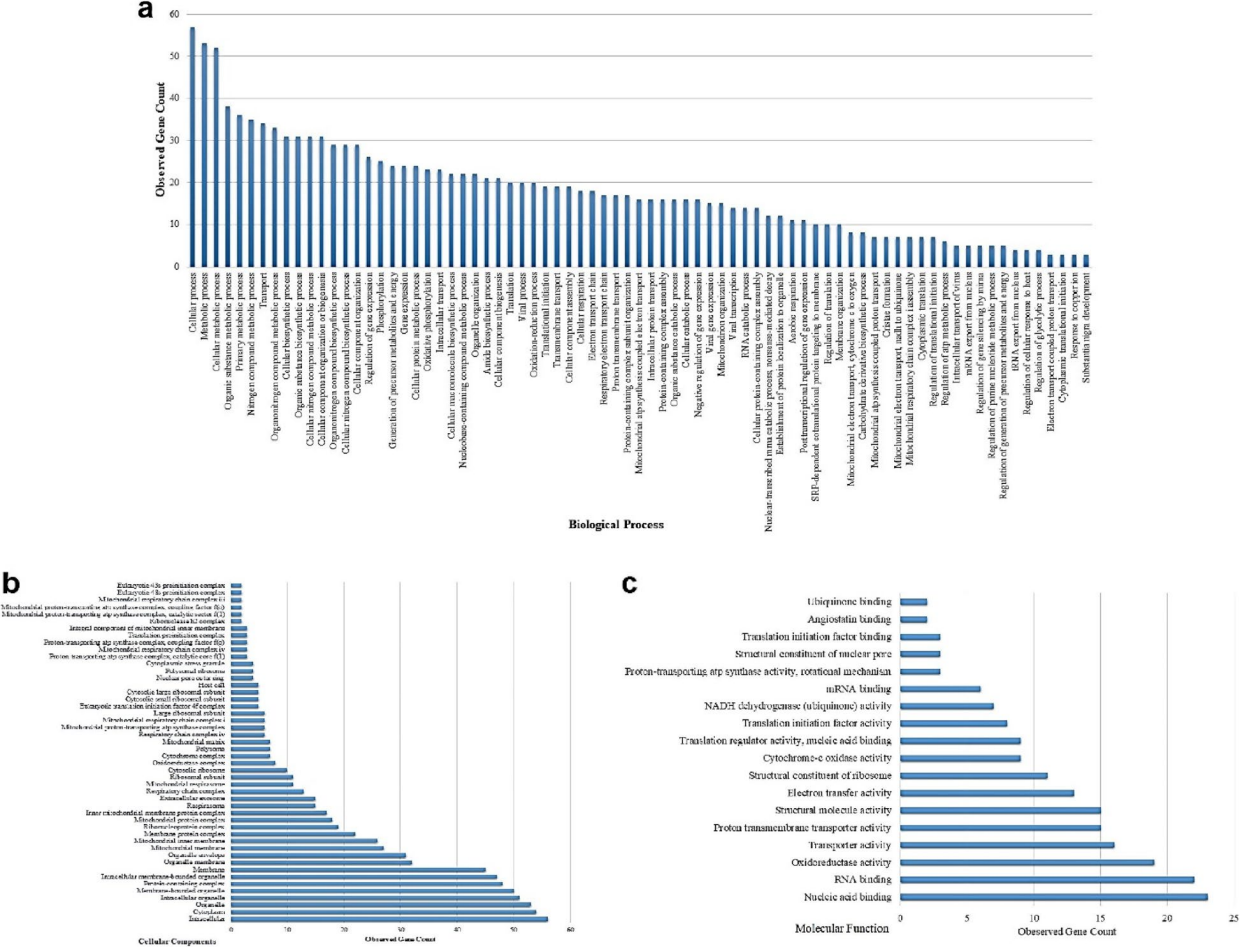
Gene Ontology enrichment analysis of the determined Subnetwork of hub genes in GC based on our previous studies [12–14] data using STRING version10 **a**) Biological Process, **b**) Cellular Components, and **c**) Molecular Function

**Fig. 4:**
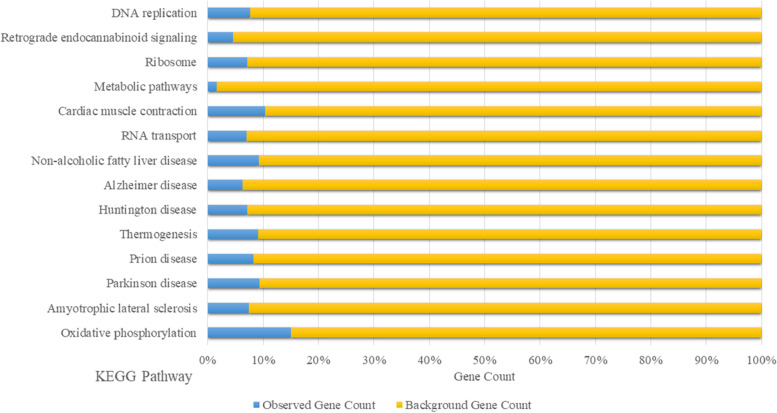
Kyoto Encyclopedia of Genes and Genomes (KEGG) pathways analysis on the hub genes in GC based on our previous studies data [12–14] using STRING version10

GO analysis is a well-known method for gene and gene products and identification of typical biological aspects of high-throughput genome or transcriptome data, including molecular function, cellular components, and biological process [24, 25]. The results of GO analysis and pathway enrichment are illustrated in Figs. 3 and 4.

The predominant (≥ 50% observed genes) GO terms found for BP are significantly enriched in cellular process, metabolic process, cellular metabolic process, organic substance metabolic process, primary metabolic process, nitrogen compound metabolic process, transport, organonitrogen compound metabolic process, cellular biosynthetic process, organic substance biosynthetic process, cellular nitrogen compound metabolic process, cellular component organization or biogenesis, organonitrogen compound biosynthetic process, cellular nitrogen compound biosynthetic process, and cellular component organization (Fig. 3a).

The predominant (≥ 50% observed genes) GO terms found for CC are intracellular, cytoplasm, organelle, intracellular organelle, membrane-bounded organelle, protein-containing complex, intracellular membrane-bounded organelle, membrane, organelle membrane, and organelle envelope (Fig. 3b).

The predominant (≥ 20% observed genes) GO terms found for BF are nucleic acid binding, RNA binding, oxidoreductase activity, transporter activity, proton transmembrane transporter activity, structural molecule activity, and electron transfer activity (Fig. 3c).

In addition, the GO results in terms of BF showed the significance of oxidoreductase activity, electron transfer activity, and proton transmembrane transporter activity which may all contribute to the mitochondrial function of ATP synthase that has been discussed in previous sections.

KEGG (http://www.genome.jp/) is a database for allocating certain pathways to groups of DEGs, therefore, linking omics data with higher-order functional data [26]. Based on KEGG pathway enrichment analysis (Fig. 4), the prominent (≥ 40% observed genes) pathways are amyotrophic lateral sclerosis, Parkinson's disease, Prion disease, Alzheimer's disease, Metabolic pathways, Oxidative phosphorylation, Thermogenesis, and Huntington's disease. Most of these pathways are related to neurodegenerative diseases [27].

### Cluster analysis of the network

One of the most important strategies for identifying the functional modules and predicting network biomarkers and complexes of proteins is cluster analysis of the biological networks. In addition, when the results of clustering are visualized, the biological network structure can be displayed. CytoCluster, which is used in this study, includes six clustering algorithms. The selection of the clustering algorithm depends on the user requirements [17]. The IPCA algorithm used in this study is density-based, identifying dense subgraphs in protein interaction networks. IPCA determines the weight of an edge by calculating the common neighbors of its connected nodes and calculates each node’s weight through a sum up of the weights of its incident edges. A node needs to have a higher weight to be regarded as a seed. At first, a seed is considered a cluster. IPCA then extends a cluster by adding vertices recursively from its neighbor regarding the nodes’ priority. Adding a node to a cluster depends on two parameters: 1) the probability of its interaction and 2) the shortest path between it and the nodes in the cluster [17]. Eleven clusters were obtained in the cluster analysis of the subnetwork. Clusters with ranks 1 to 4 were selected to be discussed in this paper. As shown in Table 3, the common pathways among all four cluster ranks include oxidative phosphorylation, Parkinson's disease, thermogenesis, prion disease, Huntington's disease, Amyotrophic lateral sclerosis, Alzheimer's disease, non-alcoholic fatty liver disease, metabolic pathways, cardiac muscle contraction, and retrograde endocannabinoid signaling. However, the Ribosome pathway is not shown only in cluster rank 4, and the DNA replication pathway is only shown in cluster rank 3. These results confirmed the common pathways involved in both neurodegenerative diseases and GC.

**Table 3:** Summary of the clusters (rank 1 to 4) resulted from the cluster analysis of the subnetwork of the overexpressed genes in Gastric Cancer plus their known neighbors based on data provided in our previous studies [12–14] using the CytoCluster App

**Cluster Rank**	**Nodes**	**Edges**	**Pathways**
**1**	33	405	Oxidative phosphorylation Parkinson disease Thermogenesis Prion disease Huntington disease Amyotrophic lateral sclerosis Alzheimer disease Non-alcoholic fatty liver disease Metabolic pathways Cardiac muscle contraction Ribosome Retrograde endocannabinoid signaling
**2**	32	391	Oxidative phosphorylation Thermogenesis Parkinson disease Prion disease Huntington disease Amyotrophic lateral sclerosis Alzheimer disease Non-alcoholic fatty liver disease Metabolic pathways Cardiac muscle contraction Ribosome Retrograde endocannabinoid signaling
**3**	30	342	Oxidative phosphorylation Thermogenesis Parkinson disease Prion disease Huntington disease Amyotrophic lateral sclerosis Alzheimer disease Non-alcoholic fatty liver disease Metabolic pathways Cardiac muscle contraction Retrograde endocannabinoid signaling Ribosome DNA replication
**4**	26	284	Oxidative phosphorylation Thermogenesis Parkinson disease Prion disease Huntington disease Amyotrophic lateral sclerosis Alzheimer disease Metabolic pathways Non-alcoholic fatty liver disease Cardiac muscle contraction Retrograde endocannabinoid signaling

KEGG pathway analyses showed that the oxidative phosphorylation (OXPHOS) pathway was considerably enriched, and the cluster analysis confirmed it.

### Promoter motif analysis of DEGs

The UFRs (1000 bp) of DEGs were analyzed to identify the conserved motifs and consensus cis-regulatory elements (CREs). The UFRs were extracted using Ensembl BioMarts, a hub for data retrieval across taxonomic space [28]. The MEME Suite web server offers an integrated portal for online finding and assessing motifs indicating features such as protein interaction domains and DNA binding sites. Motifs of transcription factors (TFs) can be compared with motifs in several common motif databases using TOMTOM, an algorithm for scanning the motif databases. Further analysis of TF motifs can be done for putative activities by association with GO terms, including BP, MF, and CC, using GOMO, which is the motif-GO term association tool [18]. Accordingly, the extracted UFR sequences were analyzed using TOMTOM [19] to find significant motifs. Subsequently, selected motifs were analyzed using GOMO [20]. Ten significant motifs were detected with lengths ranging from 20 to 24nt in length. The results of promoter analysis revealed that KLF16, MAZ, PATZ1, ZNF467, WT1, VEZF1, TBX15, SP1, SP2, and SP3 are among the most common transcription factor families having a binding site in promoters of our hub genes. The GOMO analysis for the motifs discovered by MEME identified several interesting biological functions (Table 4).

**Table 4:** The conserved motifs found in promoters of DEGs by MEME analysis. The UFRs of DEGs were analyzed to identify the conserved motifs and CREs. The extracted UFR sequences were analyzed using TOMTOM to find significant motifs, and selected motifs were analyzed using GOMO. Ten significant motifs were detected with lengths ranging from 20 to 24nt in length

**#**	**Motif**	**Transcription Factors**	**Logo**	**Top 5 Specific Predictions**
**1**	KLF16_HUMAN.H11MO.0.D	KLF16		CC transcription factor complex BP negative regulation of signal transduction MF chromatin binding MF protein homodimerization activity MF protein heterodimerization activity
**2**	MAZ_HUMAN.H11MO.0.A	MAZ		CC transcription factor complex BP negative regulation of signal transduction MF protein heterodimerization activity MF protein homodimerization activity CC dendrite
**3**	PATZ1_HUMAN.H11MO.0.C	PATZ1		CC transcription factor complex BP negative regulation of signal transduction MF protein heterodimerization activity MF protein homodimerization activity BP inner ear morphogenesis
**4**	SP1_HUMAN.H11MO.0.A	SP1		CC transcription factor complex BP negative regulation of signal transduction MF potassium ion binding BP negative regulation of neuron apoptosis BP potassium ion transport
**5**	SP2_HUMAN.H11MO.0.A	SP2		CC transcription factor complex BP negative regulation of signal transduction MF protein heterodimerization activity BP neuron fate commitment MF chromatin binding
**6**	SP3_HUMAN.H11MO.0.B	SP3		CC transcription factor complex BP negative regulation of signal transduction MF protein heterodimerization activity MF protein homodimerization activity MF chromatin binding
**7**	TBX15_HUMAN.H11MO.0.D	TBX15		CC transcription factor complex BP negative regulation of signal transduction MF protein heterodimerization activity MF protein homodimerization activity MF chromatin binding
**8**	VEZF1_HUMAN.H11MO.0.C	VEZF1		CC transcription factor complex CC dendrite MF transcription activator activity MF protein heterodimerization activity BP negative regulation of signal transduction
**9**	WT1_HUMAN.H11MO.0.C	WT1		CC transcription factor complex MF protein heterodimerization activity BP negative regulation of signal transduction CC dendrite MF potassium ion binding
**10**	ZN467_HUMAN.H11MO.0.C	ZNF467		CC transcription factor complex BP negative regulation of signal transduction MF protein heterodimerization activity MF protein homodimerization activity BP inner ear morphogenesis

Gene Ontology indicated that these motifs participated in potassium ion transport, negative regulation of neuron apoptosis, neuron fate commitment, and inner ear morphogenesis. However, involvement in the negative regulation of signal transduction was the most common biological process among all motifs. Therefore, this biological process may play an important role in GC pathogenesis. In addition, the predominant GO terms found for CC were significantly enriched in transcription factor complex and dendrite. Moreover, these motifs involved molecular functions, including chromatin binding, protein homodimerization activity, protein heterodimerization activity, potassium ion binding, and transcription activator activity. Therefore, these biological processes play an important role in the pathogenesis of GC.

### Aging effects

The age analysis revealed the presence of a few genes whose expression presents some changes considering time intervals and in particular around 60 years: MTRNR2L12, EIF4G2, and EIF4B.

## Discussion

In recent years, different investigations in the area of multi-center genomics research from gene to systems level and next-generation sequencing, clarified the various mechanisms specifically involved in the tumor progression [29]. This study used bioinformatics analysis to further investigate the genes involved in GC pathogenesis. In addition, the current study tries to shed light on changes in disease progression and comorbidities related to age.

Eleven hub genes were identified in this study, most of which were involved in mitochondrial functions. Recently, many cancer investigations have focused on the mitochondria for further elucidation of the molecular mechanisms of carcinogenesis [23]. Mitochondria have complicated and diverse functions, including cell signaling pathways involved in the oncogenesis, natural immunity, and preparation of building blocks for new cells, modulation of biosynthetic metabolism, cell death, control of the redox homeostasis and energy supply [30–32]. Different studies provided evidence about the potential correlation between the protein alterations and the number of mitochondria, as well as abnormal components and function of the mitochondria with several human cancer progression and prognosis in patients, signifying the possible functionality of mitochondria in new anti-tumor therapeutic strategies by targeting the mitochondrial proteins or metabolism [29]. However, the characteristics of the respiratory and metabolic changes in GC have not still been fully elucidated.

Although the observation of mitochondrial dysfunction in various cancers, the concept of the involvement of both mitochondrial glycolysis and metabolism in cancer cells is controversial. On the other hand, the critical role of the mitochondria in the maintenance of cancer is inevitable. Consequently, there have been several discussions and investigations about the alterations in the function of the mitochondria and their protein expression in various human cancers [23]. Based on Warburg’s observations, he suggested that the aerobic use of glucose in cancerous cells shows mitochondrial respiration impairment which might be the inherent cause of cancer [23]. Today, it has been demonstrated that genetic and molecular complications lead to an irregular proliferation of the cancer cells, which might change their biochemical metabolism, including enhancement of aerobic glycolysis, usually without any corruption in the function of the mitochondria [23, 33].

Mitochondrial quality control and biogenesis are enhanced in tumors. In addition, mutations in the enzymes of the nuclear-encoded mitochondrial tricarboxylic acid (TCA) cycle have been reported in some tumors leading to the production of oncogenic metabolites. However, negative selection is exploited for pathogenic mutations of the mitochondrial genome [23]. It has been reported that removal of the mtDNA leads to the restriction of tumorigenesis, and human cancers with mutant mitochondrial genomes are shown to be quite benign. Therefore, mitochondria have a multifunctional and critical role in the pathogenesis of cancers, and mitochondria seem to be a promising therapeutic target for cancer [23, 33, 34].

The respiratory chain, located in the mitochondrial membrane, is composed of 5 complexes, including Complex I or CxI (NADH dehydrogenase), Complex II (SDH), Complex III (cytochrome bc1), Complex IV (cytochrome c oxidase), and Complex V (ATP synthase) [23]. In our study, most of the identified hub genes are related to complexes I, IV and V. Among these genes, ATP5A1, ATP5B, ATP5D, and MT-ATP8 encode different subunits of the ATP synthase. “F1F0 ATP synthase”, also known as “mitochondrial membrane ATP synthase”, recruits a proton gradient across the inner membrane, which is generated by electron transport complexes of the respiratory chain to produce ATP from ADP. [35]. Liu et al*.* introduced ATP5A1 as one of the up-regulated genes in GC [36]. ATP5B is one of the most important subunits of ATP synthase and increases cellular ATP levels. Wang et al*.* found that high ATP5B expression in tumor tissues of GC is positively correlated with age, tumor size, the TNM stage, lymph node metastasis, and patients’ poor prognosis. They reported that ATP5B overexpression in GC cells caused ATP-promoting migration, invasion, and proliferation. In their study, an increase in MMP2 expression results in the phosphorylation of FAK, and phosphorylated AKT was observed by ATP5B overexpression in GC cells. Also, an increased level of extracellular ATP happens after ATP5B overexpression through the intracellular ATP secretion, and the FAK/AKT/MMP2 pathway is activated. Activation of the ATP5B-induced downstream pathway is activated through the P2X7 receptor. Inhibition of P2X7, FAK, AKT, and MMP2 results in the suppression of proliferation, migration, and invasion of GC cells. In conclusion, studies by Wang et al*.* showed that ATP5B involves in GC tumor progression through FAK/AKT/MMP2 pathway [37]. Therefore, ATP5B may serve as a poor prognosis biomarker and a beneficial therapeutic target for GC.

MT-ATP8, as one of the components involved in the OXPHOS pathway, is a mitochondrial gene encoding membrane subunit 8 of ATP synthase. Several genetic syndromes that contributed to mitochondrial dysfunction depicted by a reduced OXPHOS ability have been explained [38]. In addition, reduced OXPHOS in the mitochondria has been reported in many types of cancer cells which is related to either or both reduced flux in the TCA cycle and/or respiration [30]. Furthermore, MT-ATP8 was reported to have mutations in GC [39]. In the current study, we reported the genes related to the OXPHOS pathway, however, the OXPHOS reduction cannot be demonstrated based on our data, and it needs to be further investigated.

ATP5D encodes the subunit delta of the catalytic core of ATP synthase, F1. Wei et al*.* found that overexpression of PSMB10, VPS13D, NDUFS8, ATP5D, POLR2E, and HADH were correlated with adverse overall survival in acute myeloid leukemia (AML) [40].

NDUFS3, MT-ND4, and MT-ND6, as three other hub genes identified in this study, encode subunits of CxI. MT-ND6 encodes NADH dehydrogenase 6, part of CxI in the mitochondria. CxI is involved in the first step in the electron transport process, in which the electrons are transferred from NADH to ubiquinone. Electrons are subsequently thrown from ubiquinone through various other enzyme complexes leading to the preparation of energy for ATP generation [41].

Ishikawa et al*.* showed that the MT-ND6 mutation resulted in the suppression of CxI activity and induction of ROS production, leading to the stimulation of metastasis in breast and lung cancer cells [11]. There is one report for MT-ND6 in GC which is based on bioinformatics analysis [42].

MT-ND4 encodes NADH dehydrogenase 4, a part of CxI, one of the enzyme complexes involved in OXPHOS. In the whole mitochondrial genome sequencing study in GC, it has been reported that the tumor has remarkably more variants in the MT-ND4 region [39].

NDUFS3 encodes one of the iron-sulfur protein components of mitochondrial NADH: ubiquinone oxidoreductase (CxI). Recent studies in GC showed the prognostic capabilities of Nicotinamide N-methyltransferase (NNMT). They found that NNMT expression was positively connected to clinical pathologic stage, tumor size, and lymph node status in GC. Silencing the NNMT gene resulted in the inhibition of proliferation, invasion, and migration of the GC cells. These results represent the potential of NNMT as a beneficial prognostic marker of GC. It has been observed in neuroblastoma that the presence of NNMT could meaningfully reduce the death of SH-SY5Y cells, and the effects of NNMT were positively correlated with the elevated intracellular ATP content, ATP/ADP ratio, and CxI activity, as well as a decrease in the degradation of the NDUFS3 subunit of CxI [43]. There are various studies on the evaluation of the role of NDUFS3 as a part of CxI in different cancers. NDUFS3 has been suggested as a biomarker for the breast. ovarian and a few kidney cancers. However, there is no report on the evaluation of the role of NDUFS3 in gastric cancer [44].

Among the identified genes in this study, COX7A2 and COX6C, encode cytochrome c oxidase subunits. COX7A2 encodes subunit 7A2 of the cytochrome c oxidase, which is the terminal module of the respiratory chain in the mitochondrial and catalyzes the transfer of electrons from the reduced cytochrome c to oxygen. Data extracted from MALDI-MSI experiments have represented that COX7A2 expression is correlated with the survival curve in GC [45]. Elsner et al*.* showed that increased expression of COX7A2 is a characteristic feature of intestinal metaplasia in the esophagus [46].

COX6C encodes subunit 6c of the COX complex, which catalyzes the final step of the electron transfer chain. Recently, many investigations have reported the unusual level of COX6C in different cancerous and non-cancerous disease conditions such as diabetes, uterine leiomyoma, prostate cancer, melanoma tissues, breast cancer, and follicular thyroid cancer. It has been reported that the overexpression of NDUFA4 leads to substantial upregulation of the COX6C, which subsequently promotes GC cell proliferation and reduces apoptosis in these cells [47].

RPL8 and RPS16 were also identified in the present study. RPL8 encodes ribosomal L8 protein, which is one of the components of the 60S subunit. Based on a bioinformatics analysis-based study, among the genes highly expressed in one of the clusters, few were associated with ribosomal protein-encoding genes such as RPL8 [22]. RPS16 encodes ribosomal protein S16. The diseases associated with RPS16 include Diamond-Blackfan Anemia and Descending Colon Cancer [23].

The oxidoreductase activity which was considerably enriched in our GO analysis has been investigated in various cancers, including the breast, liver, gastrointestinal tract, and kidney. Also, it has been shown that Xanthine oxidoreductase (XOR) expression is negatively correlated with poor prognosis in these malignancies [48].

The OXPHOS pathway which was considerably enriched in our KEGG and cluster analyses produces energy in most cells, and as the final stage of cellular respiration, is composed of two parts: the electron transport chain (ETC) and chemiosmosis. In the ETC, the electrons are transferred from one molecule to the other, and the released energy from this transfer is applied in the formation of an electrochemical gradient. In chemiosmosis, ATP is made from the stored energy in the gradient. ATP synthase exploits the produced gradient of the proton to generate ATP after the phosphorylation of ADP. At the end of the ETC, oxygen accepts the electrons and makes water with protons [30]. Four genes identified in the current study (ATP5A1, ATP5B, ATP5D, and MT-ATP8) encode the subunits of the ATP synthase, three genes (NDUFS3, MT-ND4, and MT-ND6) encode subunits of complex I, and two genes (COX7A2 and COX6C) encode subunits of complex IV. These findings highlight the role of mitochondrial respiratory chain components and OXPHOS pathway in GC pathogenesis.

Recent studies presented that expression levels of several genes, such as NDUFB7, UQCRC2, and UQCRQ in the OXPHOS pathway, were meaningly down-regulated in GC. In addition, studies showed that the enhanced expression of the genes in this pathway, such as UQCRQ, NDUFB7, and UQCRC2, positively correlated with a better prognosis [49]. It has been also reported that defection in UQCRQ, as a subunit of complex III, can cause mitochondrial dysfunction, which is correlated with the pathogenesis of ulcerative colitis [23]. Also, it has been recently reported that a mutation in the UQCRC2 gene might lead to a deficiency of the mitochondrial complex III, a relatively rare disease [50]. Substantially lower content of UQCRC2 has also been reported in breast cancer cells compared to normal cells [51]. Downregulation of this gene has also been reported in glioma and GC [52]. According to these various investigations [23] showing the role of mitochondria in cancer, the malfunctioned bioenergetic mitochondria can be suggested as a symbol of tumorigenesis.

It has been reported that besides the role of the oncogenes and tumor suppressors in the expression control of the metabolic enzymes, mitochondrial energy metabolism is also affected by environmental conditions, and it has been shown that mitochondrial dysfunction is one of the characteristics of tumor cells [33]. Under various conditions, including mitochondrial DNA mutation or activation of the oncogenes, cancer cells have different abilities to use oxygen, and few studies are available on the identification of the strict relationship between the metabolic changes and composition and activity of mitochondrial complexes [30]. A substantial decrease in the activity of NADH dehydrogenase and complex I protein content has been reported in renal and lung cancer, and a reduction in the activity of complex I was also reported in thyroid cancer which was related to ND1 gene [30].

Association of mitochondrial dysfunctions with OXPHOS complexes content was also reported in a few studies [33]. A decrease in the ATP synthase content has been reported in renal cancers and chromophilic tumors, indicating inefficient structure and activity of the complex in mitochondria [53]. It has been suggested that low amounts of ATP synthase may have a critical role in the metabolism of cancer cells because carcinogenesis has been observed to have substantial effects on the expression of the F1-ATPase beta subunit leading to the changes in the control mechanisms of mitochondrial differentiation [54].

The switching of cellular energy production from OXPHOS by mitochondria to aerobic glycolysis, named the Warburg effect, happens in several cancer types [33]. One of the major characteristics of fast-growing cancer cells is to maintain an increased glycolysis level providing enough ATP irrespective of the existence of oxygen in the environment [49, 55]. However, the importance of this switching for GC development is poorly understood. Recently, *Feichtinger *et al*.* investigated the expression of OXPHOS complexes in GC using immunohistochemistry. They found that CxI expression was significantly reduced in the intestinal type of GC (but not the diffuse type of GC). Higher expression of complex I and II was seen in larger tumors, and higher expression of complex II and III was observed in higher grades [56]. On the other hand, Su et al*.* found that all of the identified DEGs involved in OXPHOS were down-regulated. They also reported that GC has substantially changed metabolic processes, including NADH dehydrogenase complex assembly and tricarboxylic acid cycle, consistent with our KEGG analysis results [49]. Therefore, the expression status of the OXPHOS complexes in GC needs to be further studied to elucidate the exact role of these complexes in the pathogenesis of GC.

Mitochondrial changes have been also reported to be correlated with tumor cell migration, invasion, and resistance to chemotherapy [23]. The primary suggestion that mtDNA and mitochondrial dysfunction might be involved in metastasis originated from Ishikawa et al., who reported that mitochondria transfer from more aggressive tumors into less aggressive ones led to enhanced aggressiveness [57]. Furthermore, various mutations in the components of the ETC complex I resulted in enhanced ROS levels, causing an enhanced metastatic characteristic [57, 58]. Particularly, mutations of ND4 and ND5 in breast cancer cell line [59] and ND6 in lung cancer cell line [41] have been observed to cause enhanced metastasis. Other mtDNA SNPs also changed the efficiency of metastasis [60]. In addition to serving as metabolic ‘powerhouses of the cell’, mitochondria have co-evolved with their hosts to serve as critical signaling hubs in several pathways. For example, mitochondrial signaling can influence cancer and metastasis in inflammation [61] and apoptosis [62]. Most of the identified genes in our study (ATP5A1, ATP5B, ATP5D, MT-ATP8, COX7A2, COX6C, ND4, ND6, NDUFS3 and RPS16) contribute to mitochondrial function suggesting their potential involvement in GC pathogenesis through various pathways. However, the correlation between these genes in regulating mitochondrial function and cancer pathogenesis continues to be unclear and requires to be validated by more research.

Many of the enriched pathways in our KEGG analysis were also related to neurodegenerative diseases and based on the previous studies [27], Sirutin 3 (SIRT3) is one of the common genes among these pathways. SIRT3 is a member of the sirtuin protein family known as class III histone deacetylases. Recent investigations have focused on SIRT3 due to its function in stress resistance, aging, neurodegenerative disease, and cancer. SIRT3 manages energy requests in situations, including mitochondrial metabolism. The elimination of reactive oxygen species and prevention of cancer cell development or apoptosis are the abilities of SIRT3, which highlights its critical role in cancer and various diseases, including Alzheimer's disease, amyotrophic lateral sclerosis, Parkinson’s disease, and Huntington's disease [27].

The results of pathway enrichment analysis in our study also showed that the metabolic processes are the most affected in GC. Recent investigations have focused on the elucidation of the correlation between metabolic reprogramming and the pathogenesis of cancer. Specifically, regulation of the metabolism and cancer investigation is more brought into intense attention with the development of metabolomics [63]. Metabolomics offers comprehensive knowledge of the metabolic profiles of certain cancers. It may provide an excellent tool to find biomarkers for prognosis, diagnosis, metastatic surveillance, and therapeutic sensitivity estimate. Various metabolic changes have been identified in GC, including glucose metabolism, amino acid metabolism, lipid metabolism, and nucleotide metabolism. In addition to the mentioned metabolic alterations in GC, changed levels of other metabolites, including creatinine and inositol, have also been reported in GC [63].

Our results also showed the potential significance of RNAs in GC pathogenesis, confirming recent studies focused on elucidating the role of RNAs in the pathogenesis of this cancer [64]. Using an inclusive assessment of the results from other studies, it has been demonstrated that circular RNAs (circRNAs) regulate the cellular biological behaviors in GC, including epithelial-mesenchymal transition (EMT), proliferation, migration, and invasion. In addition, circRNAs are correlated to the GC characteristics, including tumor stage, differentiation of the tumor, and metastasis. Therefore, circRNAs might be suitable to be used as prognostic or diagnostic biomarkers in GC. In addition, the circRNAs involved in GC pathogenesis can be used as targets in GC treatment [64].

By integrated bioinformatics analysis in 2022, Zhang et al. studied the stomach adenocarcinoma and introduced seven hub genes (EWSR1, ESR1, CLTC, PCMT1, TP53, HUWE1, and HDAC1) as related genes to occurrence of stomach adenocarcinoma [65]. Through a systems biology approach in 2022, Salarikia et al. reported that medications such as pantoprazole, omeprazole, imatinib, troglitazone, fostamatinib, and amiloride might be useful in GC treatment. They also found that disorders such as liver fibrosis, ovarian carcinoma, breast carcinoma, lung cancer, liver carcinoma, and prostate cancer might have correlation with gastric adenocarcinoma through certain genes, including *mt2a, fn1, hgf, col1a1, col1a2,* and *mmp2*. In their study, overexpression of signaling pathways, including extracellular matrix organization, cell division, and cell cycle, as well as downregulation of ion transport and digestion pathways were also indicated. They also reported that their identified hub genes in gastric adenocarcinoma participated in platelet activation, focal adhesion, gastric acid secretion, cell cycle and HPV infection pathways [66]. In 2018, Saberi Anvar et al. used a systems biology approach for GC biomarkers and found that cell cycle, neutropin signaling pathway and nucleotide excision are the most enriched signals. Also, they reported TP53, HNF4A and TAF1 as the most important nodes in the PPI network [67].

We also found three genes that present significant changes in the elder population: MTRNR2L12, EIF4G2, and EIF4B. MTRNR2L12 has no annotations available, so we suggest further analyzing this possible target. EIF4G2, as reported in the STRING database appears to play a role in the switch from cap-dependent to IRES-mediated translation during mitosis, apoptosis and viral infection [68]. EIF4B acts downstream of mTOR signaling in response to growth factors and nutrients to promote cell proliferation, cell growth and cell cycle progression [69]. In addition, it regulates protein synthesis through phosphorylation of EIF4B, RPS6 and EEF2K, and contributes to cell survival by repressing the pro-apoptotic function of BAD [69]. EIF4G2 (eukaryotic initiation factor 4G2) and EIF4B (eukaryotic initiation factor 4B) are both annotated as aging genes. The downregulation of eIF4B impacts the overall protein translation and the aberrant which is strongly associated with cancerous outgrowth. Moreover, the altered expression of EIF4G2, as reported in some recent work [70] is related to poor prognosis of GC. Therefore, our analysis may suggest that the expression changes of this gene with age may increase the risk of poor prognosis and suggest further investigation.

GEPIA (Gene Expression Profiling Interactive Analysis) web server is an important resource for the analysis of gene expression based on cancerous and normal samples from the GTEx and the TCGA databases and GEPIA2 is an improved version [71]. The Kaplan–Meier overall survival curves of GC patients extracted from GEPIA2 showed that high expression of the eleven hub genes identified in this study is associated with decreased overall survival rate [71]. The survival rate after 100 months for GC patients with overexpression of ATP5A1, ATP5B, ATP5D, COX6C, COX7A2, MT-ATP8, ND4, ND6, NDUFS3, RPL8, and RPS16 genes showed < 20%, 40%, > 20%, 40%, > 20%, < 40%, 30%, > 30%, > 20%, > 20%, and > 20%, respectively [71]. Accordingly, ATP5A1 overexpression is associated with the lowest survival rate in GC patients. Therefore, inhibition of ATP5A1 is suggested to be further studied as a therapeutic tool for survival improvement in GC patients that show overexpression of this gene.Although our study suggested the probable relevance between our identified genes/CREs and GC pathogenesis, little is known about the relevance of these genes/CREs to GC. The identified genes in this study might be promising diagnostic and prognostic biomarkers for GC. Moreover, clinical validation and long-term follow-up data for GC patients with a large sample size are required for investigating these genes as prognostic or diagnostic biomarkers. If these genes' diagnostic/prognostic importance is validated, they could be introduced as factors to define the treatment modality for GC. Research on targeted changes in the expression of these genes can offer novel therapeutic modalities.

## Conclusion

Eleven hub genes (ATP5A1, ATP5B, ATP5D, MT-ATP8, COX7A2, COX6C, ND4, ND6, NDUFS3, RPL8 and RPS16) in GC were identified in the current study, mostly involved in mitochondrial functions. There was no previous report on the ATP5D, ND6, NDUFS3, RPL8 and RPS16 in GC. The GO and pathway analysis showed that the metabolic processes are most affected in GC. These analyses also confirmed the potential significance of the mitochondrial role in GC pathogenesis. Although many investigations have been focused on the mitochondria further to elucidate the potential contribution of it to carcinogenesis, however, the characteristics of respiratory and metabolic changes in GC have not been fully elucidated. In addition, KEGG analysis and cluster analysis results showed that most of the affected pathways in GC were the pathways also involved in neurodegenerative diseases. KEGG pathway analyses also showed that the OXPHOS pathway was considerably enriched, and the cluster analysis confirmed it. Also, the promoter analysis results showed that negative signal transduction regulation might play an important role in GC pathogenesis. The results of this study might open new insights into GC pathogenesis. In addition, the identified genes in this study might be promising diagnostic and prognostic biomarkers or therapeutic targets for GC. This study confirms previous findings that mitochondria metabolism and its related protein alteration occur in various cancers. This work is based on bioinformatics analysis and tried to introduce important genes and pathways in GC pathogenesis. However, validation in a large number of clinical GC samples is suggested for future studies to evaluate the prognostic or therapeutic value of the identified markers in this study. Furthermore, additional studies are required to explore the inherent mechanisms and associated pathways of these genes in GC pathogenesis. Considering the complexity of cancer and the involvement of various molecular mechanisms in the pathogenesis of cancer, a single pathway cannot be used as a description of cancer pathogenesis [23].

## Data Availability

The data supporting the conclusions of the study are all provided in the manuscript.

## Abbreviations

BP: Biological process
CC: Cellular components
circRNAs: Circular RNAs
CREs: Cis-regulatory elements
DEGs: Differentially expressed genes
ETC: Electron transport chain
EMT: Epithelial-mesenchymal transition
GC: Gastric cancer
GO: Gene Ontology
IPCA: Identifying Protein Complex Algorithm
KEGG: Kyoto Encyclopedia of Genes and Genomes
MF: Molecular function
NNMT: Nicotinamide N-methyltransferase
OXPHOS: Oxidative phosphorylation
PPI: Protein–protein interaction
SSH: Suppression subtractive hybridization
TCA: Tricarboxylic acid
TFs: Transcription factors
UFRs: Upstream flanking regions
XOR: Xanthine oxidoreductase
